# Serum Inflammatory Mediators as Markers of Human Lyme Disease Activity

**DOI:** 10.1371/journal.pone.0093243

**Published:** 2014-04-16

**Authors:** Mark J. Soloski, Lauren A. Crowder, Lauren J. Lahey, Catriona A. Wagner, William H. Robinson, John N. Aucott

**Affiliations:** 1 Division of Rheumatology, Department of Medicine, Johns Hopkins University School of Medicine, Baltimore, Maryland, United States of America; 2 Division of Internal Medicine, Department of Medicine, Johns Hopkins University School of Medicine, Baltimore, Maryland, United States of America; 3 Division of Immunology and Rheumatology, Stanford University School of Medicine; Veterans Affairs Palo Alto Health Care System, Palo Alto, California, United States of America; 4 Lyme Disease Research Foundation, Baltimore, Maryland, United States of Ameica; KAIST, Graduate School of Medical Science & Engineering, Republic of Korea

## Abstract

Chemokines and cytokines are key signaling molecules that orchestrate the trafficking of immune cells, direct them to sites of tissue injury and inflammation and modulate their states of activation and effector cell function. We have measured, using a multiplex-based approach, the levels of 58 immune mediators and 7 acute phase markers in sera derived from of a cohort of patients diagnosed with acute Lyme disease and matched controls. This analysis identified a cytokine signature associated with the early stages of infection and allowed us to identify two subsets (mediator-high and mediator-low) of acute Lyme patients with distinct cytokine signatures that also differed significantly (p<0.0005) in symptom presentation. In particular, the T cell chemokines CXCL9 (MIG), CXCL10 (IP-10) and CCL19 (MIP3B) were coordinately increased in the mediator-high group and levels of these chemokines could be associated with seroconversion status and elevated liver function tests (p = 0.027 and p = 0.021 respectively). There was also upregulation of acute phase proteins including CRP and serum amyloid A. Consistent with the role of CXCL9/CXCL10 in attracting immune cells to the site of infection, CXCR3+ CD4 T cells are reduced in the blood of early acute Lyme disease (p = 0.01) and the decrease correlates with chemokine levels (p = 0.0375). The levels of CXCL9/10 did not relate to the size or number of skin lesions but elevated levels of serum CXCL9/CXCL10 were associated with elevated liver enzymes levels. Collectively these results indicate that the levels of serum chemokines and the levels of expression of their respective chemokine receptors on T cell subsets may prove to be informative biomarkers for Lyme disease and related to specific disease manifestations.

## Introduction

Lyme disease is the most frequently reported vector-borne disease in the United States, with approximately 30,000 cases reported to the CDC in 2012 [1]. The average annual rate in 2003–2005 (29.2 cases per 100,000 population) was approximately three times the *Healthy People 2010* target of 9.7 new cases per 100,000 [2], [3]. Reports likely underestimate the incidence of Lyme disease and it has been suggested that there are up to 10 times more cases than those reported to the CDC [4], [5]. The rising incidence of Lyme disease demands a more complete understanding of the disease process and particularly the disease mechanisms underlying long term outcomes of *Borrelia burgdorferi* infection such as Post-Treatment Lyme disease Syndrome (PTLDS) [6].

Lyme disease is an inflammatory disease initiated by infection with *B. burgdorferi* following a bite from an infected tick [7]. Symptoms of early acute Lyme disease can include erythema migrans (EM) with or without systemic symptoms such as fever, chills and malaise. Signs of disseminated infection may occur early or late in the disease process and can involve the skin, musculoskeletal and nervous system [8]. Pathology of EM skin lesions shows a mononuclear cell infiltrate of lymphocytes, plasma cells, and macrophages [9], [10]. Although patients will resolve the EM with or without antibiotic therapy, as the early immune response develops in response to the infection, a significant fraction of patients who receive antibiotics early in infection do not develop detectable antibodies on convalescent testing [11], [12]. In the absence of early antibiotic treatment, the host immune response does not completely eradicate the infection and a significant fraction of patients develop late-onset arthritis [7]. While the infection and late-onset arthritis can largely be controlled by antibiotic therapy, in a subset of patients, Lyme arthritis with inflammation is antibiotic-refractory, persisting up to 12 months or more. An ongoing pathologic host immune response is thought to be the primary etiology in those with persistent arthritis [13]. Circulating inflammatory markers that may be associated with this secondary complication have not been identified.

To study the immunological processes that are initiated following *B. burgdorferi* infection, we have utilized samples generated from a large cohort of Lyme disease patients that have been followed longitudinally for two years from the time of diagnosis and treatment. In this report, we measured the levels of a comprehensive panel of cytokines and chemokines to identify inflammatory mediators associated with acute Lyme disease as well as long-term outcomes of *B. burgdorferi* infection. Interestingly, mediator levels allow us to distinguish two populations of Lyme disease patients that display significant differences in the number of disease symptoms, seroconversion rates, lymphopenia and serum liver enzyme levels. Several T cell chemokines were coordinately upregulated while chemokines that drive other immune cell types were not. This work suggests that the complexity and levels of mediators present in the serum may be informative in understanding the various pathophysiological outcomes that occur in acute *B. burgdorferi* infection and that are associated with subsequent development of PTLDS.

## Results

### Identification of a Cytokine/Chemokine Signature Associated with Acute Lyme Disease

**Figure 1:**
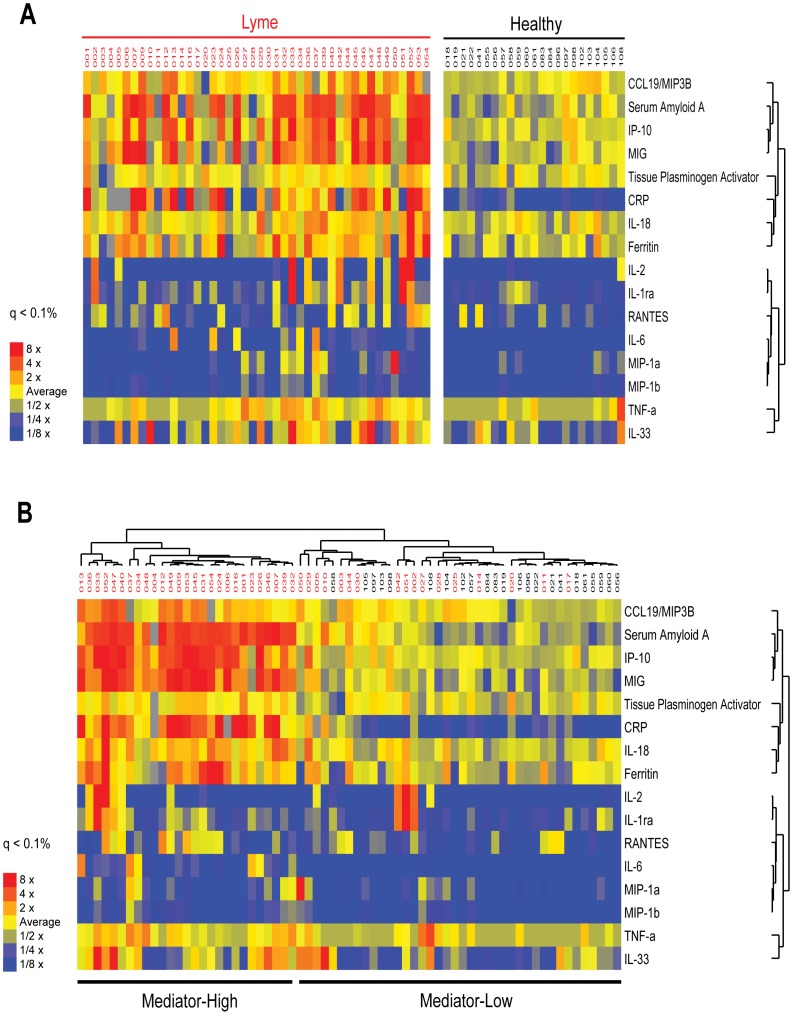
Elevated Immune Mediators in Lyme Disease. Serum samples from patients with diagnosed acute Lyme disease (n = 44, red) and healthy controls (n = 23, black) were assayed for the presence of 58 soluble mediators and 7 acute phase proteins using an optimized multiplex-based assay system. Displayed are those mediators that show significant changes (q<0.1%) in Lyme patients vs. controls. (Panel A) Results are displayed as a heat map to visualize differences in mediator levels in Acute Lyme patients relative to controls. (Panel B) Unsupervised hierarchical clustering of the results was performed, and the output displayed as a heatmap. This analysis resulted in the formation of two clusters, including a “mediator high” cluster that contains samples derived from patients with acute *B. burgdorferi* infection who exhibited elevated serum inflammatory mediators. The second “mediator low” cluster includes a subset of samples from acute *B. burgdorferi* infection as well as the matched healthy controls, both of which exhibited low levels of inflammatory mediators.

We have conducted a prospective cohort study of acute Lyme disease and post-treatment events (see Table 1). All patients enrolled in the study have untreated early Lyme disease that meets the Infectious Disease Society of America (IDSA) criteria for diagnosis [6], [11], [14]. Using samples collected from this cohort we employed a bead-based multiplex cytokine assay to measure the levels of 58 immune mediators and 7 acute phase proteins in the serum of patients with untreated early (acute) Lyme and matched controls. Displayed in figure 1A is a heat map of those mediators significantly elevated (q<0.1%) in early acute Lyme disease at the initial pre-treatment visit compared to controls. Most notable are elevated levels of several T cell chemokines (CCL19, CXCL9, CXCL10), acute phase inflammatory markers (CRP and serum amyloid A), several IL-1 cytokine family members (IL-1ra, IL-18, IL-33), inflammatory cytokines TNF-α and IL-6 and the T cell cytokine IL-2. Collectively, these cytokines and chemokines generate a novel signature that clearly distinguishes acute Lyme patients from normal controls.

**Table 1:** Cohort Characteristics.

		Cases (n = 44)	Controls (n = 23)
**Demographics**			
Sex	Male	56.8%	43.5%
	Female	43.2%	56.5%
Age		49.41±16.241 (20–75)	56.22±12.645 (22–73)
Race	White	95.5%	95.7%
	Black	2.3%	2.3%
	Other	2.3%	2.3%
Education*		15.91±2.675 (11–21)	17.74±2.435 (12–21)
Clinical Characteristics**			
Serogroup	Non-converter	35.7%	
	Converter	26.2%	
	Positive at diagnosis	38.1%	
Lesion type	Single	65.9%	
	Disseminated	34.1%	
Area (cm^2^) of primary lesion		96.73±98.96 (11.78–466.53)	
Liver enzyme tests	Normal	56.8%	
	Elevated	43.2%	
Lymphopenia status	Normal	59.1%	
	Lymphopenic	40.9%	

* = Statistically different between cases and controls (p = 0.008), but not clinically significant.

** = Early Lyme disease cohort only; these characteristics do not apply to control participants.

Displayed in figure 1B is an unsupervised hierarchical clustering of the serum mediator profiles of the same early acute Lyme disease at the initial pre-treatment visit and control patients. This analysis allows us to distinguish two patient clusters of acute Lyme disease patients. One group termed mediator-high, display-elevated levels of the T cell chemokines and inflammatory markers during acute infection described above. A second group of Lyme disease patients (mediator-low) display mediator levels that result in their clustering among normal control samples. Since all patients met IDSA guidelines for acute Lyme disease, the mediator-low group represents a subset of Lyme patients that do not exhibit significant elevations in inflammatory mediators in blood.

### Acute Lyme Disease Patient Defined Subgroups have distinct clinical features

Based on immune mediator levels during acute infection, two subgroups of Lyme patients could be distinguished. Table 2 presents a comparison of these two groups for a range of clinical outcomes. No differences in demographics, duration of illness or the distribution of single versus multiple erythema migrans lesions were noted between the two groups. However, significant differences were noted in the number of symptoms (p<0.005), absolute lymphocyte levels (p = 0.002), liver enzyme tests (p = 0.027) and the presence of detectable anti-Borrelia antibodies (p = 0.021). Lyme disease patients displaying high mediator levels at the initial pre-treatment visit (acute disease) had higher rates of seroconversion, but also had greater rates of lymphopenia and elevated liver enzyme.

**Table 2:** Acute Lyme Disease patient subsets defined by circulating mediators versus clinical phenotypes.

		Acute Lyme Mediator High n = 27 mean (SD)	Acute Lyme Mediator Low n = 17 mean (SD)	Significance
**Sex**	Female	44%	41.2%	NS
	Male	55.6%	58.8%	
**Lymphocyte number**		1.07 (0.41) ×10^3^ µl	1.58 (0.63) ×10^3^ µl	p = 0.002
**Lymphopenia Status**	Lymphopenic	70.4%	29.4%	p = 0.013
	Non-lymphopenic	29.6%	70.6%	
**Liver Enzyme Group** a	High liver enzyme	61.5%	23.5%	p = 0.027
	Normal liver enzyme	38.5%	76.5%	
**Serology** b	Seropositive	77.8%	40%	p = 0.021
	Seronegative	22.2%	60%	
**EM Presentation**	Single Lesion	66.7%	64.7%	NS
	Disseminated Lesions	33.3%	35.7%	
**Number Symptoms Pre-treatment**		8.45 (3.45)	4.15 (1.95)	p<0.0005
**Illness Duration (days)**		6.89 (4.48)	12.59 (11.65)	NS

Table 2 shows group differences based on the heat map generated using SAM (see Figure 1). No demographic differences between groups were seen. Categorical variables were compared using Fisher's Exact tests, while continuous variables were compared using unpaired t-tests. Lymphocyte values given are mean (standard deviation), while other values given are a percentage of the respective subset. Number of symptoms pre-treatment is defined as the number of symptoms reported by the patient during structured interview by the principle investigator (JNA) or study staff (LAC). Illness duration is defined as the period of time between first sign or symptom of disease and presentation for treatment and enrollment in the study.

a Acute Lyme Mediator High n = 26; Acute Lyme Mediator Low n = 17;

b Acute Lyme Mediator High n = 27; Acute Lyme Mediator Low n = 15.

### T Cell Chemokine Levels are Selectively Elevated in the Acute Phase of Lyme Disease

**Figure 2:**
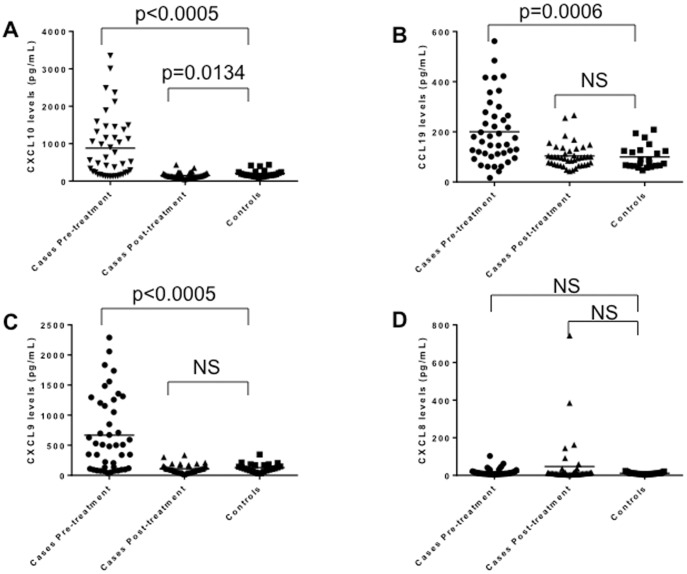
Chemokine levels in Lyme disease before and after treatment. Displayed are the levels of the chemokines CXCL10 (Panel A), CCL19 (Panel B), CXCL9 (Panel C) and CXCL8 (Panel D) measured in the serum of Lyme patients (n = 44) pre-treatment (acute disease) and post-treatment (4 weeks following diagnosis) as compared to healthy controls (n = 23). Horizontal bars represent the medians for each sample group.

A striking feature of the cytokine/chemokine profile was the upregulation of T cell specific mediators. Displayed in figure 2A–D are the measured levels of the chemokines CXCL9 (MIG), CXCL10 (IP-10), CCL19 and CXCL8 (IL-8) in sera obtained from patients at the time of their initial diagnosis of acute Lyme disease (pre-treatment), 4 weeks following diagnosis and treatment (post-treatment) and matched controls. The T cell chemoattractants CXCL9, CXCL10 and CCL19 are significantly elevated in serum during the acute infection but largely return to normal levels following treatment, resolution of the erythema migrans and recovery. Levels of CXCL9, CXCL10 and CCL19 are completely concordant with one another (Table 3). In contrast, the neutrophil chemotactic factor CXCL8 is not elevated during acute infection or at other observed time points (Figure 2D). Furthermore, the measured levels of CCL11 (eotaxin-1), CXCL1 (GROa), CCL2 (MCP-1), CCL7 (MCP-1), CCL3 (MIP-1a), CCL4 (MIP1b), CCL5 (RANTES) and CXCL12 (SDF-1a) are not significantly different as compared to the levels in matched controls (data not shown). Therefore in acute Lyme disease there appears to be a selective and coordinate elevation of T cell chemo-attractants.

**Table 3:** Correlation Analysis of Key Mediators.

Mediators	R value	Significance
CXCL9 vs. CXCL10	0.690	p<0.0005
CCL19 vs. CXCL10	0.629	p<0.0005
CCL19 vs. CXCL9	0.725	p<0.0005
CRP vs. SAA	0.373	0.016
IL-6 vs. CRP	0.268	NS
IL-6 vs. SAA	0.094	NS

Table 3 shows the relationship between acute, pre-treatment levels of key immune mediators of early Lyme disease as discussed in this paper. Pearson correlations were used for all analyses. A significant correlation can be seen between CXCL10, CXCL9 and CCL19. CRP shows a significant positive correlation with Serum Amyloid A (SAA). IL-6 does not correlate with either CRP or SAA.

### Innate Serum Inflammatory Markers are Up-Regulated during the Acute Lyme Disease

**Figure 3:**
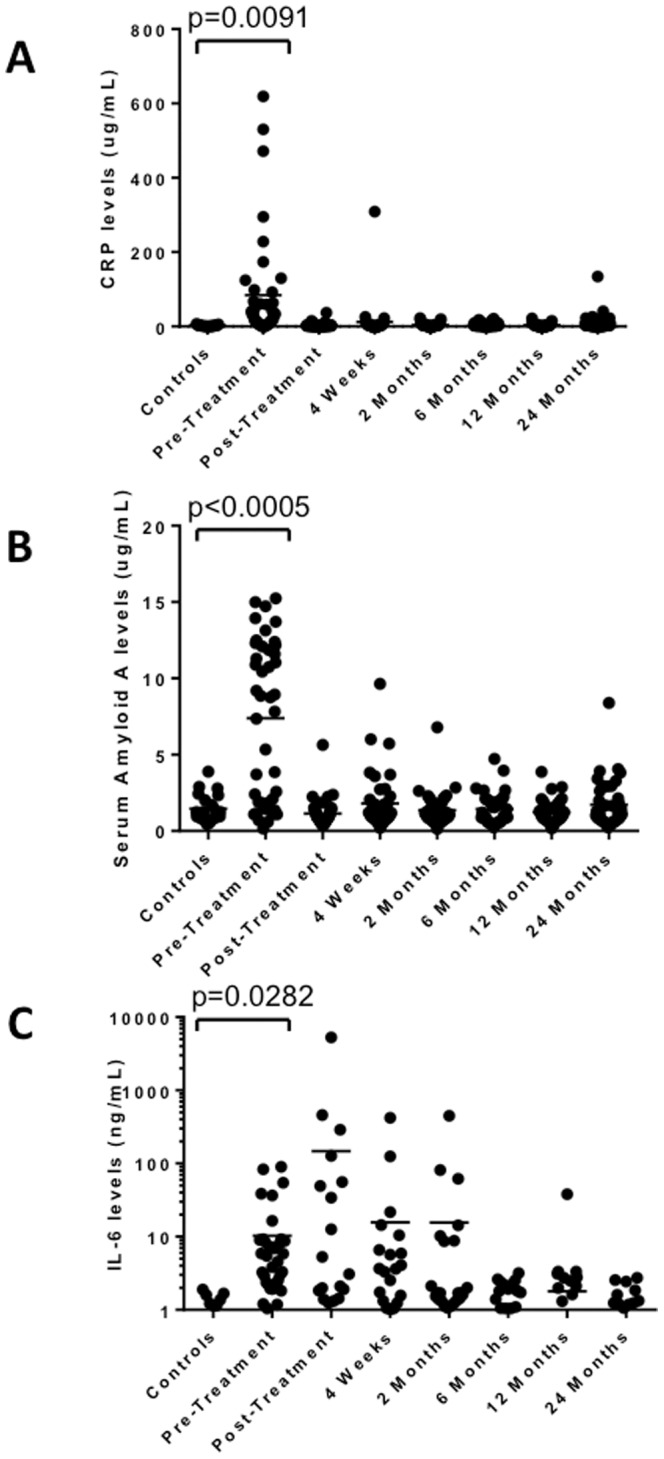
Elevated Inflammatory Mediators in Lyme Disease. Serum levels of CRP (panel A), Serum Amyloid A (Panel B) and IL-6 (Panel C) were measured in healthy controls (n = 23) and at various times during the course of diagnosis and treatment in Lyme patients (n = 44). CRP, Serum Amyloid A and IL-6 levels are significantly elevated at diagnosis (pre-treatment). CRP and Serum Amyloid A levels return to control levels after treatment while IL-6 levels persist.

As revealed in the global serum profiling shown in figure 1, the cytokine IL-6 and the innate immune acute phase factors C-reactive protein (CRP) and serum amyloid A (SAA) are elevated during acute Lyme disease. A more detailed analysis of the levels over time is presented in Figure 3. Increased levels of CRP (p = 0.0091), SAA p<0.0005) and IL-6 (p = 0.0282) were seen during the acute phase. CRP and SAA levels return to normal control levels following treatment and remain so throughout the follow-up period. IL-6 levels remain elevated and only return to normal levels months after infection and treatment. As is the case for the T cell chemokines, serum CRP and SAA significantly correlated with one another (Table 3, p = 0.016). Surprisingly, IL-6, a known inducer of CRP and SAA did not correlate with these acute phase proteins (p = 0.09 and 0.54 respectively, Table 3).

### Chemokine and Inflammatory markers: correlation with liver function abnormalities

T cell chemokine levels in acute Lyme patient sera, while clearly elevated, do show heterogeneity with a subset displaying levels similar to controls (Figure 2A–C). A similar case can be made for the inflammatory biomarkers CRP and serum amyloid A (Figure 3). This heterogeneity suggests that individual chemokine and/or inflammatory markers may correlate with clinical parameters.

The erythema migrans (EM) lesion is the primary site of inflammation in acute Lyme disease and it can vary in size as well as number. This lesion is characterized as the site of active bacterial growth and the accumulation of immune inflammatory cells that is dominated by T cells [9], [15], [16]. The chemokines CXCL9 and CXCL10 are present at high levels within the EM lesion likely produced by fibroblasts and endothelial cells in an interferon-γ dependent manner [15]–[17]. Based on this we reasoned that the levels of CXCL9 and/or CXCL10 might correlate either with the size or degree of dissemination of the EM lesion. Within the Lyme cohort, a significant proportion of patients had disseminated lesions and the size of the lesions was varied (Table 1). Surprisingly, when we compared these variables with the levels of serum CXCL9 and CXCL10 there was no statistical association (Figure S1 and Table S1).

**Figure 4:**
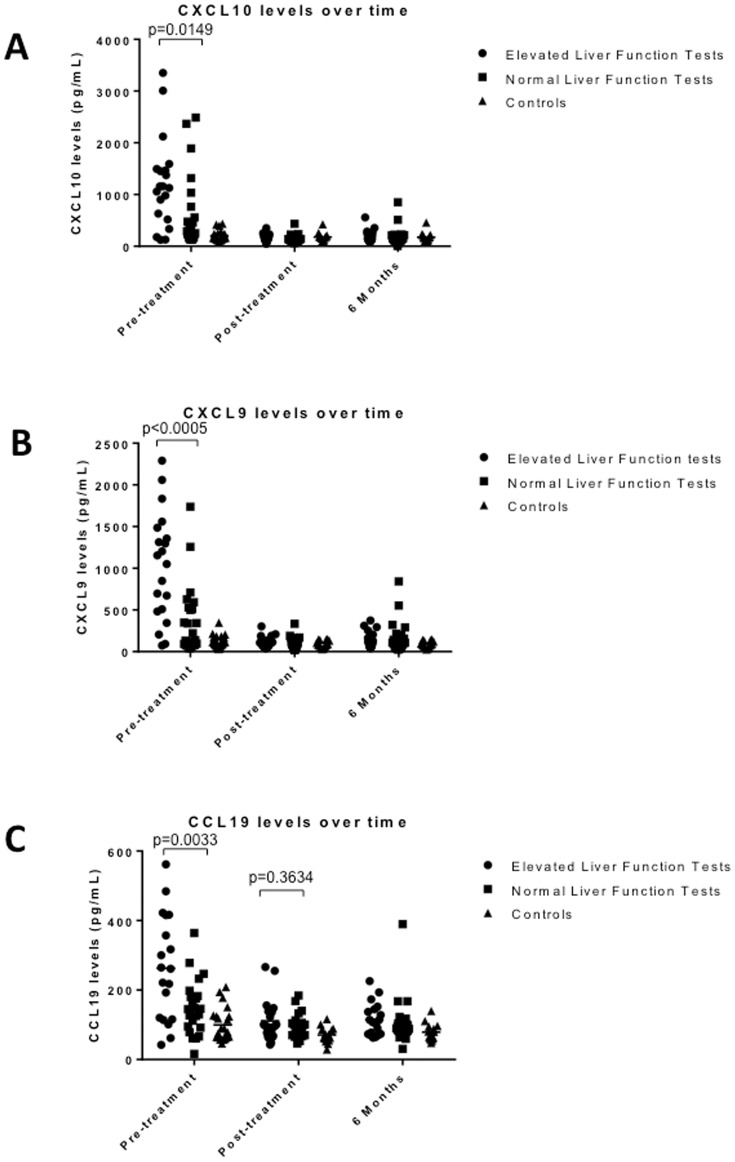
Chemokine levels are associated with elevated liver function tests. Lyme patients were separated into two populations based on normal (n = 24) vs. elevated liver enzyme tests (n = 20) and the levels of CXCL10 (Panel A), CXCL9 (Panel B) and CXCL10 (Panel C) compared at three visits (pre-treatment, post-treatment, and 6 months) relative to healthy controls (n = 23). All three chemokines are significantly different between Lyme patients with high liver function tests and those with normal liver function tests at the pre-treatment visit. This difference was not observed at later time points.

Previous studies have also described changes in liver enzyme levels during acute Lyme disease [18], [19]. This pattern is also observed in the current cohort of Lyme disease patients (Table 1). When we compared the levels of serum CXCL9/CXCL10 among the acute Lyme patients with high liver enzyme levels, there was a significant association of high CXCL9/CXCL10 and CCL19 levels with elevated liver enzymes in the acute phase of Lyme disease (Figure 4).

**Figure 5:**
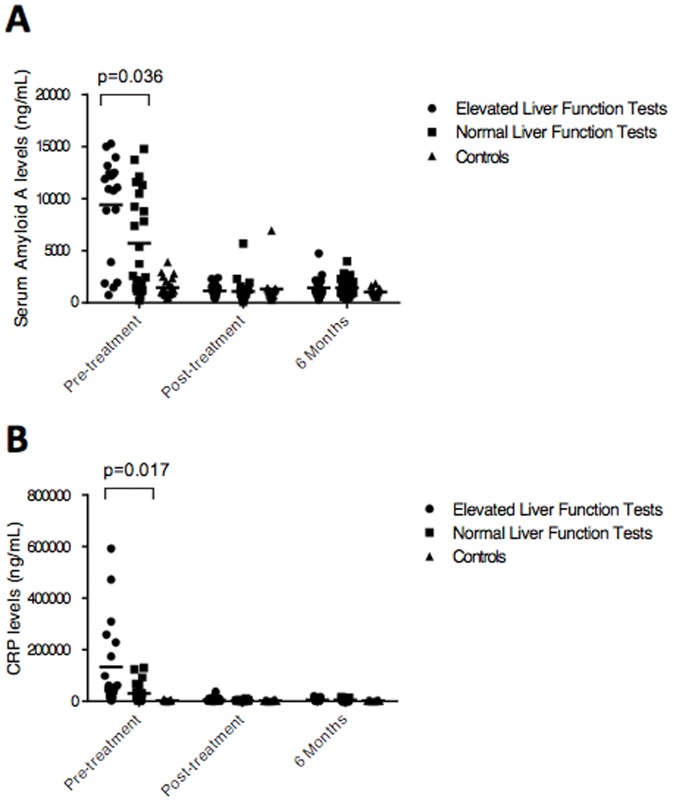
Serum Amyloid A levels are associated with elevated liver function tests. Lyme patients were separated into two populations based on normal (n = 24) vs. elevated liver enzyme tests (n = 20) and the levels of Serum Amyloid A (Panel A) and CRP (Panel B) were compared at multiple time points relative to healthy controls (n = 23). Serum amyloid A (p = 0.036) and CRP (p = 0.017) levels are significantly different between Lyme patients with high liver function tests at the pre-treatment visit. Both groups are significantly different from controls at (p<0.0005), but are not different from each other or controls, at both the Post-treatment and 6 Month follow-up visits. There is no significant difference in CRP levels between high and normal liver function groups, however both are significantly different from controls (p<0.0005).

The finding that liver enzymes and acute inflammatory markers are elevated in acute Lyme disease suggested that there these two markers may be related. As shown in Figure 5, CRP and SSA but not IL-6 (data not shown) levels correlate with liver enzyme levels.

### High T cell Chemokine Levels are associated with seroconversion

In this patient cohort, 35.7% of Lyme patients fail to test positive either at diagnosis or through seroconversion following antibiotic treatment (Table 1). This is consistent with previous studies that demonstrated that a significant fraction of Lyme patients that exhibit EM along with other symptoms of infection do not seroconvert when assayed by the current two-tiered testing [11], [12]. When we examined whether various elevated mediator levels could be correlated with seroconversion status, a significant association was observed with elevated CXCL9 and CXCL10 but not CCL19 levels (Table 4). When other clinical parameters such as duration of illness or size/dissemination of EM lesions (data not shown) were analyzed, there was no association with seroconversion status.

**Table 4:** Serostatus versus T Cell Chemokine Levels.

	Serology Negative	Serology Positive	Significance
**CXCL10 <800 pg/mL**	13	10	p = 0.003
**CXCL10 ≥800 pg/mL**	2	17	
**CXCL9 <350 pg/mL**	11	7	p = 0.004
**CXCL9 ≥350 pg/mL**	4	20	
**CCL19 <200 pg/mL**	11	15	NS
**CCL19 ≥200 pg/mL**	4	12	

Cutoffs in early Lyme disease cases were created for CXCL10, CXCL9, and CCL19 based on being higher or lower than controls. The columns show differences between those who are sero-positive at either time of diagnosis or immediately following treatment and those who are negative at both time points for these three biomarkers. CXCL10 and CXCL9 show differences in the association between those who are sero-positive and sero-negative (p = 0.003 and p = 0.004, respectively). There is no statistical difference in the association between serogroups for CCL19.

### CXCR3 expressing T cells are decreased in Lyme disease and co-relate with CXCL9/CXCL10 levels

**Figure 6:**
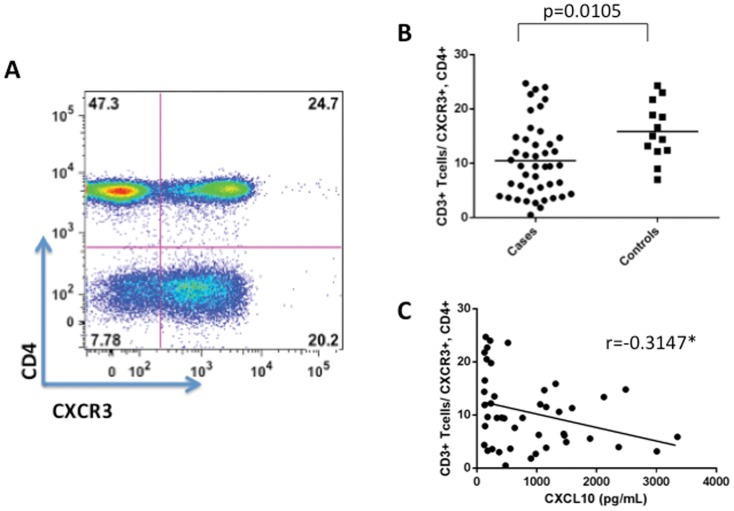
CXCR3 Expressing CD4^+^ T cell levels correlate with serum CXCL10. Panel A: CXCR3 expressing CD4^+^ T cells were detected using polychromatic flow cytometry. Displayed is a representative plot. Panel B: The frequency of CXCR3^+^CD4^+^ T cells among total CD4^+^ PBMCs were determined in Lyme patients prior to treatment (cases, n = 44) and healthy controls (n = 23). Levels of CXCR3^+^CD4^+^ T cells were lower in cases vs. controls at pre-treatment (p = 0.0105). Panel C. The levels of blood CXCR3^+^CD4^+^ T cells were negatively correlated CXCL10 serum levels in pretreatment early Lyme disease cases (p = 0.0375).

The chemokines CXCL9 and CXCL10 bind to a common receptor CXCR3 expressed largely on T cells [20]. We reasoned that high serum levels of these chemokines may drive T cells into inflamed tissues and as a result levels of CXCR3 expressing T cells may be altered during acute Lyme disease. Levels of CXCR3^+^ CD4 T cells were determined by polychromatic flow cytometry (Figure 6A), and found to be significantly lower in the blood of patients with acute Lyme disease versus controls (Figure 6B). In addition, serum levels of CXCL10 (Figure 6C) and CXCL9 (data not shown) were inversely related to the frequency of CXCR3^+^ CD4^+^ T cells in the peripheral circulation.

## Discussion

Utilizing a multiplex-based assay for 65 immune and inflammatory mediators, a clear coordinated cytokine/chemokine signature was identified that distinguished patients with acute Lyme disease from normal non-inflammatory controls. Furthermore, variation in this signature allowed us to define at least two groups of Lyme disease patients with clear differences in the levels of key mediators. Interestingly, these subgroups of Lyme patients had distinct disease characteristics including number of symptoms, lymphopenia, elevated liver function and rate of seroconversion. At this time we cannot determine if this signature is acute Lyme specific or more reflects a general inflammatory signature. However, a similar analysis of serum from patients with Rheumatoid Arthritis identified a distinct cytokine signature that included Eotaxin, Il-12p40 and Rantes, mediators that were not elevated in our Lyme disease cohort (46–49).

CXCL9, CXCL10 and CCL19 are three prominent chemokines that were elevated in our cohort of acute Lyme disease patients. All three mediators are coordinately elevated and return to baseline control levels following treatment and resolution of the EM. Previous work has shown elevated levels of CXCL9 and CXCL10 within the EM skin lesion and in the sera of patients with early acute Lyme disease, as well as in the synovial fluid and tissue of patients with Lyme arthritis [16], [17], [21]–[23]. Similar to our observations, the levels of serum CXCL9 and CXCL10 can vary among acute Lyme disease patients and levels correlate with severity of disease [23]. Elevated levels of CXCL9 and CXCL10 are found in a number of Th1 driven immune inflammatory settings including autoimmune disorders and viral, bacterial and protozoan infections [20], [24], [25].

The chemokines CXCL9 and CXCL10 are produced by macrophages and non-immune cells within inflamed tissues in an interferon-dependent manner. These chemokines bind to the chemokine receptor CXCR3 expressed largely on antigen activated T cells [20]. The primary site of inflammation and bacterial replication in early acute Lyme disease is thought to be the skin EM lesion. Previous work has shown that this site expresses high levels of CXCL9 and CXCL10 and CXCR3^+^ T cells [15], [17]. Our finding that high levels of serum CXCL9 and CXCL10 levels are associated with lymphopenia and correlate with lower levels of blood CXCR3+ T cells supports a model where infection–induced tissue inflammation and chemokine production drives the recruitment of activated effector T cells from the blood into the site of infection. Therefore it was surprising when we observed no correlation of CXCL9 or CXCL10 levels with the size or extent of the EM. Interestingly, high levels of CXCL9 and CXCL10 were closely related with the extent of liver involvement as measured by blood liver enzyme levels. In mouse models, the liver is a well-defined site for *B. burgdorferi* dissemination [26]. Also, liver function can vary among acute Lyme patients [18], [27], [28]. These observations suggest that *B. burgdorferi* induced liver involvement can, in part, be driving the serum levels of CXCL9 and CXCL10. Consistent with this is the finding that CXCL9 and CXCL10 recruits T cells in chronic liver diseases and that CXCR3^+^ effector T cells accumulate in the liver of *B. burgdorferi* infected mice [26], [29]. Interestingly, CXCL10 levels correlate with severe liver damage levels in hepatitis-C infected patients [30], [31]. Whether this is the case for Lyme disease requires further study.

To our knowledge this is the first observation that the serum levels of the chemokine CCL19 is elevated in acute Lyme disease. Previous work has shown an increase in the cerebrospinal fluid (CSF) in subjects with Lyme neuroborreliosis where CXCL19, along with CXCL13, is proposed to play a role in B cell recruitment [32]. CCL19 is a ligand for CCR7 and plays an important role in the homing of B and T lymphocytes and dendritic cells to the lymph node to facilitate cellular interactions essential for the generation of an effective immune response [33], [34]. The coordinated elevation of CXCL9, CXCL10 and CCL19 in acute Lyme disease is consistent with an ongoing host immune response in the draining lymph nodes accompanied by the generation of *Borrelia* - reactive effector T cells and their migration into the site of infection. These conditions would be predicted favor the generation of an effective antibody response and, indeed seroconversion is significantly associated with elevated levels of CXCL9 and CXCL10.

The detection of a subgroup of acute Lyme patients that display low levels of immune mediators in the blood (mediator-low) could represent a set of immunologically hyporesponsive individuals or patients that have immunologically cleared the infection for which inflammation has subsided. The finding that both mediator groups have similar duration of illness and identical erythema migrans presentation together with the observation that the mediator–low group is also enriched for seronegative patients argues against but does not fully exclude the latter possibility. Of note the mediator low group is heterogeneous in how Lyme patients cluster with controls suggesting that indeed there may be multiple mechanisms underlying the mediator low group. The failure to seroconvert is a well-known feature of antibiotic treated early Lyme disease but the underlying cause is not understood [11], [12]. It is also unlikely that the non-seroconverting Lyme disease patients were missed due to a delayed response because in the study design patients with a seronegative test result at diagnosis were re-tested following treatment and remained negative. This argues that the mediator low group represents a subgroup of Lyme patients that develop a diminished immune response that leads, in some cases, to poor antibody production.

In a well-developed inbred mouse model, infection with *B. burgdorferi* led to disrupted germinal center formation, delays in the generation of long lived plasma cells and a weak, largely IgM antibody responses [35]. It was proposed that this outcome may be part of a *B. burgdorferi* evasion strategy to delay or avoid clearance. Although this was not tested in the above model, it is possible that *B. burgdorferi* strains may vary in evasive capabilities and be capable of stimulating a range of antibody responses. It is reasonable to speculate that the seronegative subjects within the mediator-low group may have been infected with a highly evasive *B. burgdorferi* strain. This is supported by the finding that chemokine (CXCL9 and CXCL10) and cytokine levels can vary in Lyme disease patients depending on the genotype of the infecting *B. burgdorferi* [21]. Alternatively, and perhaps more likely, the host genetic environment may play a role as TLR-1 polymorphisms are linked to chemokine (CXCL9 and CXCL10) and cytokine levels [23].

CRP is a short pentraxin produced by the liver and functions as a fluid phase pattern recognition molecule [36]. SAA is a serum lipoprotein that can recognize bacteria by interacting with outer membrane proteins [37], [38]. CRP and SAA are synthesized by hepatocytes and IL-6 has been identified as a strong stimulator of CRP and SAA production [36], [37], [39], [40]. Infection with *B. burgdorferi* clearly stimulates the coordinated production CRP and SAA along with IL-6 during the acute stage of Lyme disease. Therefore it was surprising that the levels of serum CRP and SAA reactants correlate poorly with serum IL-6. This implies that IL-6 independent events may be driving production. Alternatively, the increases in CRP, and SAA could reflect the localized production of IL-6 in the liver, possibly as a site of infection, inflammation and/or tissue injury in the early acute Lyme phase [41]. This latter scenario is supported by the association of SAA and CRP levels with elevated serum liver enzymes (Figure 5A).

The finding that IL-6 levels remain elevated in some patients through the latter stages of Lyme disease was surprising. This suggests that in a subset of patient diagnosed with acute Lyme individuals, following treatment, there is an ongoing inflammatory event driving IL-6 production. The liver is likely not the site of inflammation as CRP and SAA levels return to normal post-treatment. One possibility is that there is residual antigen or infection in some treated Lyme disease patients but this is a controversial area. Evidence from mouse and primate models supports either the persistence of live bacteria or antigens after antibiotic treatment following Lyme borreliosis [42]–[44]. Convincing validation of these studies in the human setting is not available. Nevertheless, our observations suggest that there is an ongoing process that is selectively driving IL-6 production, the mechanistic basis of this awaits further study. Future studies need to examine if there is any relationship of persistently elevated IL-6 levels or other inflammatory markers with long-term outcomes such as PTLDS.

The analysis of chemokine and cytokine levels in the serum of Lyme disease patients has allowed us to define at least two subsets of Lyme patients which have distinct disease phenotypes differing in number of symptoms, extent of liver involvement, lymphocyte levels and seroconversion status. It is possible that the inflammatory mediator profiles identified may prove valuable as biomarkers of Lyme disease activity. Moreover, these chemokines likely identify immune pathways that are involved in the resolution of Lyme disease and as such may be potential therapeutic targets.

## Materials and Methods

### Patient Cohort

The serum and PBMC samples used have been generated as part of a prospective cohort study with age- and sex-matched controls enrolled from 2008–2013. This study includes a well-defined cohort of patients with acute Lyme disease enrolled from a Lyme endemic area of the mid-Atlantic United States. Only patients with untreated, confirmed early Lyme disease manifesting an active EM skin lesion at the time of enrollment, as defined by CDC case criteria are eligible [6], [11], [14]. Patients with a history of prior Lyme disease or the presence of confounding preexisting medical conditions associated with prolonged fatigue, pain or neurocognitive symptoms are excluded [45]. Controls are nonhospitalized age- and sex-matched and have no prior history of Lyme disease or any exclusionary medical conditions including lack of inflammatory disorders. The enrolled Lyme disease patients are followed from the time of acute infection longitudinally for a period of 2 years for a total of 7 study visits. The matched controls are followed for 2 years across 4 study visits. At each study visit, extensive clinical data and biological specimens are collected (see below).

### Clinical data assessment

At the pre-treatment study visit, a standard Complete Blood Count (CBC) and Comprehensive Metabolic Panel (CMP) are drawn and performed at an internal laboratory. In addition to these clinical tests, an additional SST tube is drawn to be sent to an outside, commercial laboratory for Lyme serology testing. Serology results are determined following the CDC's two-tier testing algorithm measuring both IgM and IgG, with time of symptom onset being determined by a structured interview with the patient at the pre-treatment study visit [6], [11], [14]. For those patients who are negative according to the two-tier serology at the first study visit, a subsequent serology is drawn at the second study visit after antibiotic treatment and sent to the same commercial laboratory for testing.

At each study visit, patients are given a physical exam, a structured interview of twenty signs and symptoms of disease, and undergo a blood draw. At the first visit, a measurement of the EM is taken and recorded.

### Cytokine/Chemokine Assays

We performed multiplex analysis of 58 cytokines/chemokines and 7 acute phase markers using the Bio-PlexTM bead array system as recommended by the manufacturer and using previously described optimized assay protocols [46]–[49]. Data processing was performed using Bio-Plex manager software version 4.4.1 and serum concentrations were interpolated from standard curves for each respective cytokine. This protocol and data generated were MAIME compliant and were deposited in the Gene Expression Omnibus Repository (accession number GSE55815).

### Flow Cytometry

Peripheral blood mononuclear cells (PBMCs) were isolated from fresh heparinized blood using Ficoll Hypaque. Monoclonal antibody reagents specific for CD3 (UCHT1), CD4 (RPA-T4), CD8 (SK1) and CXCR3 (IC6) were purchased from Becton Dickenson. PBMCs were first incubated with unlabeled human IgG to block nonspecific binding followed by incubation with fluorescent tagged monoclonal reagents. PBMCs were washed and polychromatic flow cytometry performed using a FACSAria instrument (Becton Dickinson, San Jose, CA). Lymphocytes were gated using forward and side scatter and the data analyzed using FlowJo software (Tree Star).

### Statistical Analysis

For descriptive analyses of the multiplex biomarker data as continuous variables, data was analyzed using the log of the “ratio to average”. The log of the ratio to average was calculated by setting all values less than 1 pg/mL to 1 pg/mL, and then calculating the log base 2 of [(value)/(average value in the cohort)]. The “ratio to average” values were input into SAM (Significance Analysis of Microarrays Version 4.0) [50]. The SAM output was sorted based on false discovery rates (FDRs, represented by the q value) in order to identify mediators with the greatest differences in levels between patient subgroups. We used hierarchical clustering software Cluster 3.0 to arrange the SAM results according to similarities among mediator levels, with no markers weighted in Figure 1A, and with weighting of CCL19 and Serum Amyloid A was used in the clustering for Figure 1B. The clustered results were displayed using Java Treeview (Version 1.1.5r2).

Categorical variables were analyzed using Fisher's Exact or Chi-square statistics. A standard ANOVA or unpaired t-test was used on two or more group comparisons for continuous variables. Pearson correlations were used for linear comparisons. For longitudinal continuous variables, a repeated measures ANOVA was performed. All statistical calculations were performed with SPSS software (IBM Corporation v 21).

### Ethics Statement

All human subject studies in this manuscript were reviewed and approved by the Johns Hopkins School of Medicine Institutional Review Board (protocol NA_00011170). All subjects recruited in the study were adults and all were provided written informed consent.

## Supporting Information

Figure S1 **Lack of correlation of CXCL10 levels and size of erythema migrans.** The size of early Lyme disease patients' (n = 44) erythema migrans lesions (area = cm^2^) is compared with levels of serum CXCL10. No significant correlation was observed.
(TIFF)

Table S1 **Erythema migrans (EM) presentation and CXCL10 values.** A fisher's exact test was done examining at the association between those Lyme patients with a single erythema migrans and those with disseminated lesions versus a cutoff of CXCL10. There is no significant association between these variables (p = 0.759).
(DOCX)
