# Supplementary material for: Serum Inflammatory Mediators as Markers of Human Lyme Disease Activity
Source: PLoS One. 2014 Apr 16;9(4):e93243. doi: 10.1371/journal.pone.0093243 (PMC3989169; doi:10.1371/journal.pone.0093243)
Supplement: Table S1 — Erythema migrans (EM) presentation and CXCL10 values. A fisher's exact test was done examining at the association between those Lyme patients with a single erythema migrans and those with disseminated lesions versus a cutoff of CXCL10. There is no significant association between these variables (p = 0.759). (DOCX) [file pone.0093243.s002.docx]

**Supplemental Table 1. Erythema migrans (EM) presentation and CXCL10 values***

|  |  | Single EM |  | Multiple EM |
| --- | --- | --- | --- | --- |
| CXCL10 < 800 pg/mL |  | 17 |  | 8 |
| CXCL10 ≥ 800 pg/mL |  | 12 |  | 7 |

*A fisher’s exact test was done looking at the association between those Lyme patients with a single erythema migrans and those with disseminated lesions versus a cutoff of CXCL10. There is no significant association between these variables (p=0.759).
